# Validating mitochondrial electron transport chain content in individuals at clinical high risk for psychosis

**DOI:** 10.1038/s41598-019-49180-3

**Published:** 2019-09-03

**Authors:** Abbie Wu, Tania Da Silva, Maya Jacobson, Abanti Tagore, Nittha Lalang, Michael Kiang, Romina Mizrahi, Ana C. Andreazza

**Affiliations:** 1 Department of Pharmacology & Toxicology, University of Toronto, Toronto, Ontario Canada; 2 Research Imaging Centre, Centre for Addiction and Mental Health, Toronto, Ontario Canada; 3 Department of Psychiatry, University of Toronto, Toronto, Ontario Canada; 4 Institute of Medical Science, University of Toronto, Toronto, Ontario Canada; 5 Campbell Family Mental Health Research Institute, Centre for Addiction and Mental Health, Toronto, Ontario Canada

**Keywords:** ELISA, Predictive markers

## Abstract

Altered mitochondrial electron transport chain function has been implicated in the pathophysiology and etiology of schizophrenia. To date, our previously published study (i.e. first cohort) is still the only study to demonstrate that mitochondrial electron transport chain is not altered in white blood cells from individuals at clinical high risk for psychosis. Here, we aimed to replicate our previous findings with an independent set of samples and validate the levels of mitochondrial complex I-V content in individuals at clinical high risk for psychosis. We demonstrated that the second cohort (i.e. validation cohort) expressed similar results as the first cohort. We combined the first cohort study with the second cohort and once more validated a lack of differential levels in mitochondrial complex I-V content between the two groups. In addition, we were able to validate a correlation between complex III content and prodromal negative symptom severity when the two cohorts studies were combined. Additionally, a correlation between complex V content and prodromal disorganization symptom severity was found when the two cohorts were combined. In conclusion, our results showed that dysfunction of the mitochondrial electron transport chain is not detected in peripheral blood mononuclear cells of individuals in the putative prodromal stage of schizophrenia.

## Introduction

Schizophrenia (SCZ) is a debilitating disease of the brain that significantly affects an individual’s quality of life. Studies have shown that with earlier intervention, there is better disease outcome^1^. Many studies have implicated mitochondrial dysfunction in the pathophysiology and etiology of SCZ^2^. Genetic, post-mortem, peripheral, and imaging studies have reported differences in mitochondrial and mitochondrial-related gene expression, altered electron transport chain activity, and changes in energy metabolism between non-psychiatric controls and SCZ patients^3–8^. However, research on mitochondrial function has been sparse in regard to the putative prodromal stage of SCZ. Previously, we conducted the first study investigating peripheral levels of mitochondrial electron transport chain complex in individuals at clinical high risk for psychosis (CHR) compared to non-psychiatric individuals (CTL)^9^. We demonstrated that in peripheral blood mononuclear cells (PBMCs), mitochondrial complex I-V content did not differ in individuals with CHR compared to CTL^9^. Further, we identified a negative correlation between mitochondrial complex III content and negative prodromal symptom severity, suggesting that mitochondrial complex III dysfunction might be associated with negative prodromal symptoms, warranting further studies^9^.

The lack of difference on mitochondrial electron transport chain complexes is also supported by a post-mortem brain tissue study that showed no change in complex I activity in SCZ patients^10^. Moreover, another post mortem brain study has also showed no changes in the expression of nuclear messenger RNA that code for mitochondrial proteins in SCZ patients^11^. However, these results are contradicted by many peripheral studies in SCZ patients that have shown increases or decreases in mitochondrial complex function. A study that explored mitochondrial complex activity in PBMCs of stable chronic SCZ patients showed decreased complex I activity but no change in complex II or III activity^12^. Another study that examined the platelets of SCZ individuals in acute exacerbation found increased mitochondrial complex I activity but no change in complex IV activity^13^. Furthermore, another study found increased mitochondrial complex I activity in the platelets of patients in acute exacerbation and chronic active state, but decreased complex I activity in patients in residual state^14^. A possible explanation for the contradiction is that in the first cohort we focused on CHR individuals, and not individuals that have already developed SCZ; which is the main focus of the literature in relation to mitochondrial dysfunction^9^ (Table 1).

**Table 1:** Peripheral mitochondrial electron transport chain alterations in schizophrenic and clinical high risk individuals.

Diagnosis		Results
SCZ	Ben-Shachar *et al*.^13^ SCZ individuals in acute exacerbation (n = 77)	Increased mitochondrial complex I activity (p < 0.001) in platelets No change in mitochondrial complex IV activity in platelets
	Dror *et al*.^14^ SCZ in acute exacerbation (n = 37) SCZ in chronic active state (n = 50) SCZ in residual state (n = 26)	Increased mitochondrial complex I activity in platelets of patients in acute exacerbation (p < 0.0001) and chronic active state (p < 0.0001) Decreased mitochondrial complex I activity in platelets of patients in residual state (p < 0.002)
	Gubert *et al*.^12^ Stable chronic SCZ individuals (n = 18)	Decreased mitochondrial complex I activity (p = 0.02) in PBMCs No change in mitochondrial complex II and III activity in PBMCs
CHR	Da Silva *et al*.^9^ (n = 26)	No change in mitochondrial complex I-V content

The purpose of our first cohort was to examine changes in peripheral mitochondrial content in CHR individuals and to explore correlations to clinical symptoms. Our first cohort, to the best of our knowledge, was the first to examine mitochondrial complex content in individuals with CHR^9^. In this study, additional CTL and CHR participants were recruited in an attempt to validate the initial results. We used the same protocols, biological sample collection methods, and statistical analysis as the previously published study^9^, in order to replicate the study design. In this study, we replicated the lack of difference in mitochondrial complex I-V content between individuals with clinical high-risk for psychosis and non-psychiatric controls. Samples were combined to increase power of analysis and results confirm the lack of difference in mitochondrial complex I-V content between individuals with clinical high-risk for psychosis and non-psychiatric controls. This suggests that protein content of mitochondrial electrons transport chain in peripheral white blood cells might not be a good biological target (i.e. biomarker) for assessment or investigation of risk in the psychosis prodrome. Other markers such as mitochondrial DNA content or mRNA expression of mitochondrial subunits remain to be fully investigated to understand whether these methods could detect mitochondrial dysfunction in CHR population.

## Results

### Validation cohort

#### Demographic information

Demographic data from the second cohort is shown in Table 2. There were no statistically significant differences in age, sex, body mass index (BMI), nicotinamide nucleotide transhydrogenase (NNT) levels, and current tobacco use between the CTL and CHR groups. There were significantly more individuals in the CHR group who tested positive for cannabis use as compared to the CTL group (χ^2^(1, 33) = 3.35, p = 0.07). There were 6 CHR currently using antipsychotics with two individuals taking Aripiprazole (2.0 mg and unspecified amount), three individuals taking Quetiapine (50 mg, 200 mg, and 6.25 mg), and one individual taking Risperidone (0.5 mg).

**Table 2:** Demographic and clinical information of the validation cohort.

		Non-psychiatric Controls	Clinical High Risk		
		(n = 14)	(n = 19)		
Age (years), SD		21.29 ± 2.64	21.63 ± 3.50	*U* = 131	*p* = 0.95
Sex	Male	9	15	χ^2^ = 0.87	*p* = 0.35
	Female	5	4		
BMI, SD		24.42 ± 4.82	23.32 ± 7.38	*U* = 97.5	*p* = 0.20
NNT levels (MFI), SD		1282 ± 878.8	1735 ± 1006	*t* = 1.35	*p* = 0.19
Current Drug Use	Tobacco	0	1	χ^2^ = 0.76	*p* = 0.38
	Cannabis	0	4	χ^2^ = 3.35	*p* = 0.07
Antipsychotic Use		0	6		
SOPS, SD	Total		38.74 ± 14.60		
	Positive		11.37 ± 3.86		
	Negative		13.16 ± 6.59		
	Disorganization		6.42 ± 3.78		
	General		7.79 ± 4.35		
RBANS, SD	Total	88.55 ± 13.12	94.17 ± 12.45		
	Immediate memory	95.00 ± 17.62	93.78 ± 11.55		
	Visuospatial memory	83.09 ± 16.11	89.06 ± 15.23		
	Language	88.73 ± 20.53	99.06 ± 22.03		
	Attention	99.73 ± 16.77	102.50 ± 15.11		
	Delayed memory	91.91 ± 13.12	94.78 ± 10.11		

Abbreviations: SD, standard deviation; BMI, body mass index; NNT, nicotinamide nucleotide transhydrogenase; MFI, median fluorescence intensity; SOPS, Scale of Psychosis-risk Symptoms, RBANS, Repeatable Battery for the Assessment of Neuropsychological Status.

#### Mitochondrial complex I-V content

**Figure 1:**
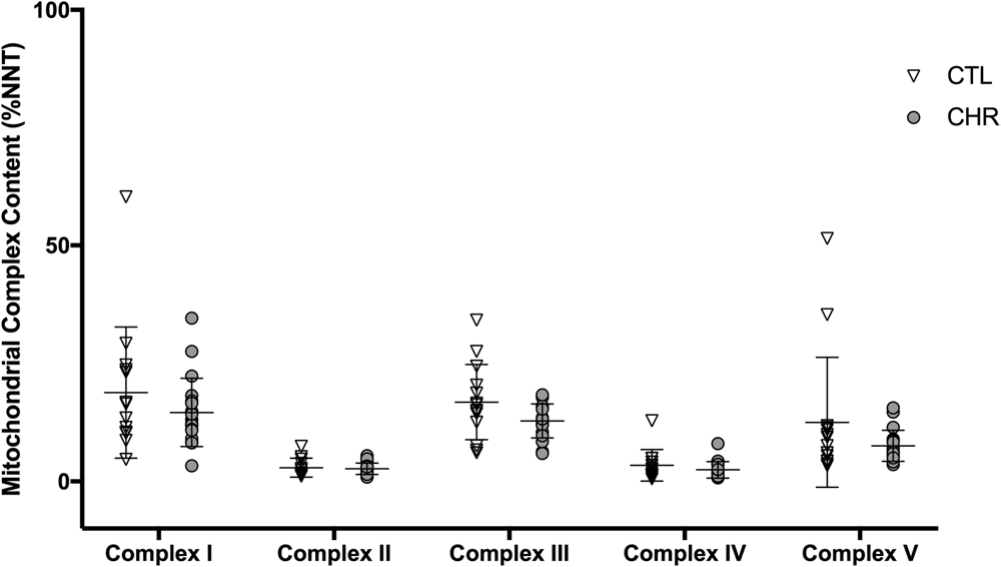
Mitochondrial complex I-V content in the clinical high risk (CHR) group compared to non-psychiatric controls (CTL); reported in %NNT content in the validation cohort.

We confirmed in these independent samples that there were no significant differences between CTL and CHR in the level of mitochondrial complex I (F(1,27) = 0.17, p = 0.68), complex II (F(1,27) = 0.06, p = 0.81), complex III (F(1,27) = 1.51, p = 0.23), complex IV (F(1,27) = 0.18, p = 0.68), and complex V (F(1,27) = 0.21, p = 0.65) content (Fig. 1). After controlling for age, sex, BMI, antipsychotic use, tobacco use, and cannabis use there were still no significant differences between CTL and CHR in the protein levels of mitochondrial complex I (F(1,21) = 2.31, p = 0.14), complex II (F(1,21) = 2.46, p = 0.13), complex III (F(1,21) = 2.11, p = 0.16), complex IV (F(1,21) = 1.62, p = 0.22), and complex V (F(1,21) = 2.11, p = 0.16) content. There were no significant correlations between mitochondrial complex I-V content and SOPS in the CHR group and RBANS assessments in both CTL and CHR groups (p > 0.05).

### Combining first cohort samples and validation cohort to investigate sample size effect

#### Demographic information

Demographic data of both studies combined is shown in Table 3. There were no statistically significant differences in age, sex, BMI, and NNT protein levels between the CTL and CHR groups. There were 8 current tobacco users in the CHR group; which differs significantly from the CTL group (Χ^2^(1, N = 75) = 5.97, p = 0.015), and 6 current cannabis users; which differs significantly from the CTL group (Χ^2^(1, N = 75) = 4.35, p = 0.037). Combined, there were 11 CHR individuals currently using antipsychotics. In addition to the 6 individuals taking antipsychotics from the current study, the 5 individuals from the first cohort study include one individual currently taking 75 mg of Quetiapine, three individuals taking Risperidone (one individual taking 0.5 mg, and the other two individuals taking 1.0 mg), and one individual taking 5.0 mg of Aripiprazole.

**Table 3:** Demographic and clinical information after combining the first study and the validation cohort.

		Non-psychiatric Controls	Clinical High Risk		
		(n = 30)	(n = 45)		
Age (years), SD		21.27 ± 2.303	20.84 ± 2.688	*U* = *577*	*p* = *0*.*287*
Sex	Male	14	30	Χ^2^ = 2.97	*p* = *0*.*085*
	Female	16	15		
BMI, SD		24.42 ± 4.82	23.32 ± 7.38	*U* = *597*.*5*	*p* = *0*.*406*
NNT levels (MFI), SD		1282 ± 878.8	1735 ± 1006	*U* = *617*	*p* = *0*.*536*
Current Drug Use	Tobacco	0	8	Χ^2^ = 5.97	*p* = *0*.*015*
	Cannabis	0	6	Χ^2^ = 4.35	*p* = *0*.*037*
Antipsychotic Use		0	11		
SOPS, SD	Total		36.68 ± 11.51		
	Positive		11.59 ± 3.62		
	Negative		11.77 ± 5.62		
	Disorganization		4.61 ± 2.93		
	General		8.07 ± 4.04		
RBANS, SD	Total	87.19 ± 13.42	91.18 ± 13.79		
	Immediate memory	94.93 ± 16.12	94.57 ± 13.99		
	Visuospatial memory	82.00 ± 18.97	87.41 ± 13.64		
	Language	86.48 ± 18.90	90.41 ± 22.87		
	Attention	98.67 ± 15.28	100.73 ± 16.34		
	Delayed memory	90.33 ± 12.89	94.05 ± 10.02		

Abbreviations: SD, standard deviation; BMI, body mass index; NNT, nicotinamide nucleotide transhydrogenase; MFI, median fluorescence intensity; SOPS, Scale of Psychosis-risk Symptoms, RBANS, Repeatable Battery for the Assessment of Neuropsychological Status.

#### Mitochondrial complex I-V content

**Figure 2:**
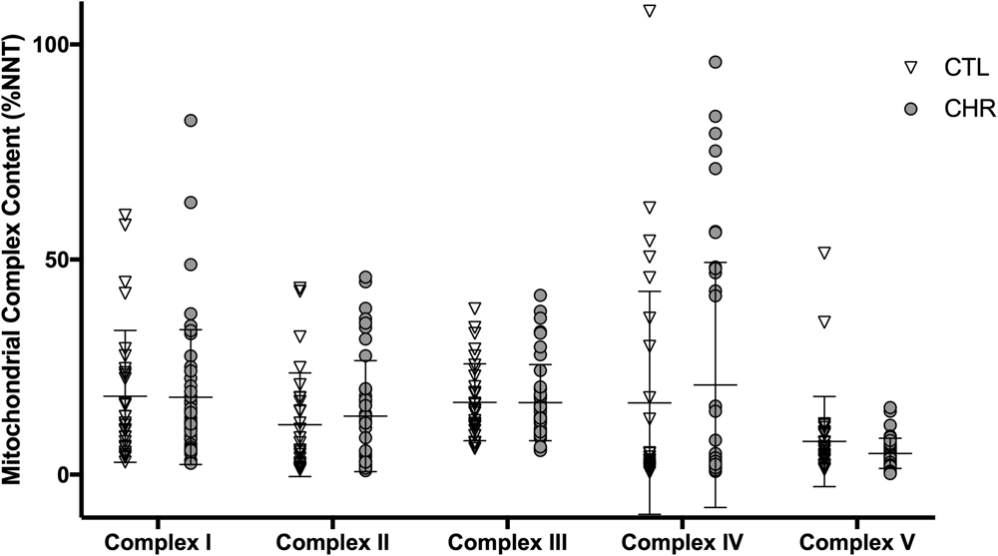
Mitochondrial complex I-V content in the clinical high risk (CHR) group compared to non-psychiatric controls (CTL); reported in %NNT content when combining the first study and the validation cohort.

Here we further validate that there were no significant differences between CTL and CHR in the levels of mitochondrial complex I (t(73) = 0.090, p = 0.93), complex II (U = 554, p = 0.48), complex III (t(73) = 0.019, p = 0.99), complex IV (U = 591, p = 0.78), and complex V (t(73) = 1.22, p = 0.23) content (Fig. 2). After controlling for age, sex, BMI, antipsychotic use, tobacco use, and cannabis use there were still no significant differences between CTL and CHR in the levels of mitochondrial complex I (F(1, 67) = 1.49, p = 0.23), complex III (F(1,67) = 0.56, p = 0.56), and complex V (F(1, 67) = 3.61, p = 0.062) content. An ANCOVA could not be performed on the levels of mitochondrial complex II and IV as the data were non-normally distributed. In the CHR group, complex III content was inversely correlated with SOPS negative symptom severity score (r(43) = −0.38, p = 0.009) (Supplementary Fig. S1) and SOPS total symptom severity score (r(43) = −0.30, p = 0.047) (Supplementary Fig. S2); and complex V content was positively correlated with SOPS disorganization severity score (r(43) = 0.50, p = 0.001) (Supplementary Fig. S3). Aside from the correlation between complex III content and SOPS total symptom severity, results survived after Bonferroni correction for multiple comparisons.

## Discussion

This study successfully validates the results of our first published cohort^9^ reporting no statistically significant difference in mitochondrial complex I-V content between CTL and CHR; both in an independent sample set and also with an increased number of samples. These results may seem to be contradictory to previously published literature that focuses on differences in mitochondrial activity between patients with fully developed SCZ in comparison to CTL groups and not CHR^12–14^.

Mitochondrial complex I activity in platelets is shown to be increased in individuals in acute exacerbation of SCZ compared to CTL^13,14^ and decreased in individuals in residual state SCZ^7^. Furthermore, a study which examined activity of mitochondrial complexes in the PBMCs of individuals with SCZ in stable chronic condition found decreased mitochondrial complex I activity^12^. However, these studies also found no changes in platelet mitochondrial complex IV in individuals in acute exacerbation of SCZ^13^; and PBMC mitochondrial complex II and III in individuals with stable chronic SCZ^12^. The alterations in mitochondrial complex activity seen in the literature differ from the results of this study; and it is likely due to the difference in groups studied. The current literature related to psychosis and peripheral mitochondrial complex activity focuses on individuals that have already developed psychosis^12–14^; however the population studied in our first and second studies are individuals that are at risk to develop psychosis. These individuals are in the putative prodromal state of schizophrenia^15^ with an expected 30% transition risk to a full-blown psychotic episode in the first two years^16^. A longitudinal test following CHR individuals that have converted to full psychosis would be required to see how peripheral mitochondrial complex content is altered during disease progression as compared to CTL. This may provide an explanation into the alteration in mitochondrial complex activity seen in the literature; determining if the alterations seen in the periphery are present from the early stages of SCZ, or if it is a consequence of disease progression.

When the studies were combined, we were able to validate a negative correlation between mitochondrial complex III content and negative prodromal symptom severity that was seen in the published first study. Negative symptoms are characterized by apathy and generally low motivation, which could be associated with lower energy levels. However, it is a speculative comment, as we have not measured adenosine triphosphate (ATP) levels. Moreover, the literature still in its infancy with one report demonstrating the coenzyme Q10, an antioxidant, is capable to reverse anhedonia induced by chronic restrain stress in rats^17^. We were unable to validate the negative correlation between mitochondrial complex V content and the RBANS attention subscale in CTL or when the CHR and CTL groups were combined, that was seen in the published first cohort (Supplementary Figs S4 and S5). Lastly, a new positive correlation was found between mitochondrial complex V content and the SOPS disorganization symptoms score when the first and second cohorts were combined. Although a non-significant positive correlation appeared in both the first cohort and validation studies independently, they were exploratory analyses only.

The lack of differences seen in the levels of mitochondrial complexes content between CTL and CHR in this study may indicate that changes in mitochondrial complex content in the putative prodromal population are not yet systemic, or may only be present in the brain; as alluded by higher levels of lactate in the brain^5,8^. Additionally, longitudinal studies are warranted to understand whether mitochondrial dysfunction is a consequence of disease progression, and if its systemic manifestations are only noted in late stages of the illness^12–14^. In summary, our results from our first study and this validation study support that mitochondrial electron transport chain dysfunction is not present in PBMCs of individuals in the putative prodromal stage of schizophrenia.

## Methods

### Participants and sample collection

Previously, the first cohort study conducted by Da Silva *et al*.^9^ recruited 42 participants; which included 16 non-psychiatric controls (CTL) and 26 CHR individuals. Demographic data for the previously published first study can be found in Da Silva *et al*.^9^. For this second study, there were 33 additional participants recruited; including 14 CTL and 19 CHR individuals. CHR individuals were identified through the Focus on Youth Psychosis Prevention (FYPP) Clinic at the Centre for Addiction and Mental Health (CAMH) after a diagnosis of prodromal risk syndrome as determined by the Criteria of Prodromal Syndromes (COPS). Clinical status and severity of prodromal symptoms were assessed with the Structured Interview for Psychosis-Risk Syndromes (SIPS), scale of psychosis-risk symptoms (SOPS)^18,19^. SOPS contains four different subscales of prodromal symptom severity: positive symptoms, disorganization symptoms, and general symptoms^18,19^. Methods of participant inclusion, assessing for prodromal symptoms and cognition, drug screening, and sample collection can be found in our first cohort^2^ study. We have followed the same methods to ensure replication. This study was approved by the Research Ethics Board at the Centre of Addiction and Mental Health. Procedures were thoroughly explained to participants before they provided written and informed consent. All procedures were performed in accordance with the guidelines and regulations established by ICH GCP E6 and Health Canada.

### Mitochondrial complex I-V content

Methods of evaluating mitochondrial complex I-V content can be found in our first cohort^2^ study. We have followed the same methods to ensure replication.

To account for variable mtDNA content between individuals, results were reported as a percentage of each participants’ levels of nicotinamide nucleotide transhydrogenase (%NNT). NNT is a nuclear encoded protein in the inner mitochondrial membrane that is closely related to oxidative phosphorylation^20^. Thus, it serves as an internal reference for each participant.

### Statistical analyses

Methods of statistical analyses were replicated from the first study^2^. Non-normally distributed data was log transformed before analysis. In addition to the tests performed as described by Da Silva *et al*.^9^; the effect of group (CTL vs CHR) on mitochondrial complex I-V content was assessed with MANOVA and a MANCOVA. BMI, age, sex, acute cannabis use, acute tobacco use, and antipsychotic use were included as covariates in the MANCOVA. Methods of statistical analyses were replicated from Da Silva *et al*.^9^ to examine associations between mitochondrial complex I-V content, and cognition and prodromal symptoms. As a further validation, a MANOVA and a MANCOVA were also performed on data from the previously published first study; with the same covariates added to the MANCOVA. Results confirmed non-significant differences between groups for each mitochondrial complex (Supplementary Table S1). Data from both the previously published first study and the second study were then combined. The effect of group on mitochondrial complex I-V content was assessed with independent t-tests and ANCOVAs for normally distributed data, and Mann-Whitney U tests for non-normally distributed data. BMI, age, sex, acute cannabis use, tobacco use, antipsychotic use, and study (first or second study) were included as covariates in the ANCOVAs. Methods of statistical analyses were replicated from Da Silva *et al*.^9^ to examine associations between mitochondrial complex I-V content, and cognition and prodromal symptoms. A Kruskal-Wallis test was performed to compare mitochondrial complex I-V content in CTL and CHR between the first study, the second study, and the studies combined. All statistical analyses were performed on SPSS (version 20.0) and graphics were created using GraphPad Prism (version 7.0a, 2016).

## Supplementary information

Supplementary Figures

## Data Availability

The datasets generated and analyzed during the current study are available from the corresponding author on reasonable request.
