# Supplementary material for: Validating mitochondrial electron transport chain content in individuals at clinical high risk for psychosis
Source: Sci Rep. 2019 Sep 3;9:12695. doi: 10.1038/s41598-019-49180-3 (PMC6722130; doi:10.1038/s41598-019-49180-3)
Supplement: Supplementary file 1 — Supplementary Figures [file 41598_2019_49180_MOESM1_ESM.docx]

**Validating mitochondrial electron transport chain content in individuals at clinical high risk for psychosis**

Abbie Wu^1#^, Tania da Silva^2#^, Maya Jacobson^2^, Abanti Tagore^2^, Nitha Lalang^2^, Michael Kiang^3^ Romina Mizrahi^2,3,4,5^*, Ana C. Andreazza^1,4^.

Table S1: MANOVA and MANCOVA results for Da Silva et al. 2018, comparing mitochondrial complex I-V content in individuals with clinical high risk for psychosis compared to non-psychiatric controls. The MANCOVA added the covariates of age, sex, BMI, current cannabis use, current tobacco use, and current antipsychotic use

|  | MANOVA | | | MANCOVA | | |
| --- | --- | --- | --- | --- | --- | --- |
|  | df | F-value | p-value | df | F-value | p-value |
| Complex I | 1,40 | 0.36 | 0.55 | 1,34 | 0.006 | 0.94 |
| Complex II | 1,40 | 1.19 | 0.28 | 1,34 | 0.48 | 0.49 |
| Complex III | 1,40 | 0.74 | 0.39 | 1,34 | 0.48 | 0.49 |
| Complex IV | 1,40 | 0.45 | 0.51 | 1,34 | 0.04 | 0.85 |
| Complex V | 1,40 | 0.6 | 0.44 | 1,34 | 0.6 | 0.44 |


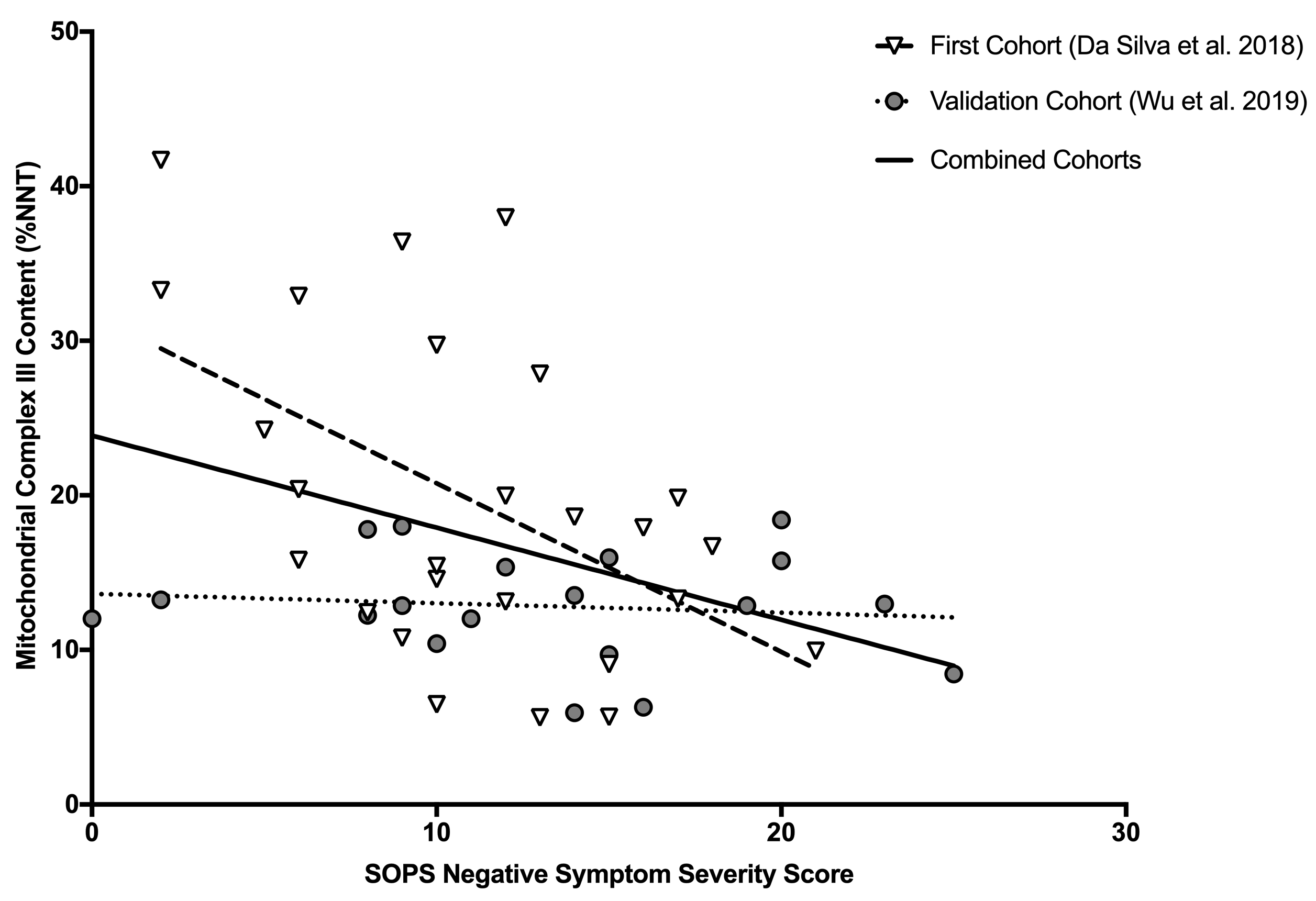


Figure S1: The comparison of bivariate correlation analyses between the first cohort, the second cohort, and the combination of the cohorts; examining the relationship between mitochondrial complex III content and SOPS negative symptom severity score in the clinical high risk for psychosis group. The effect sizes are |r| = 0.51, |r| = 0.11, and |r| = 0.38 respectively.


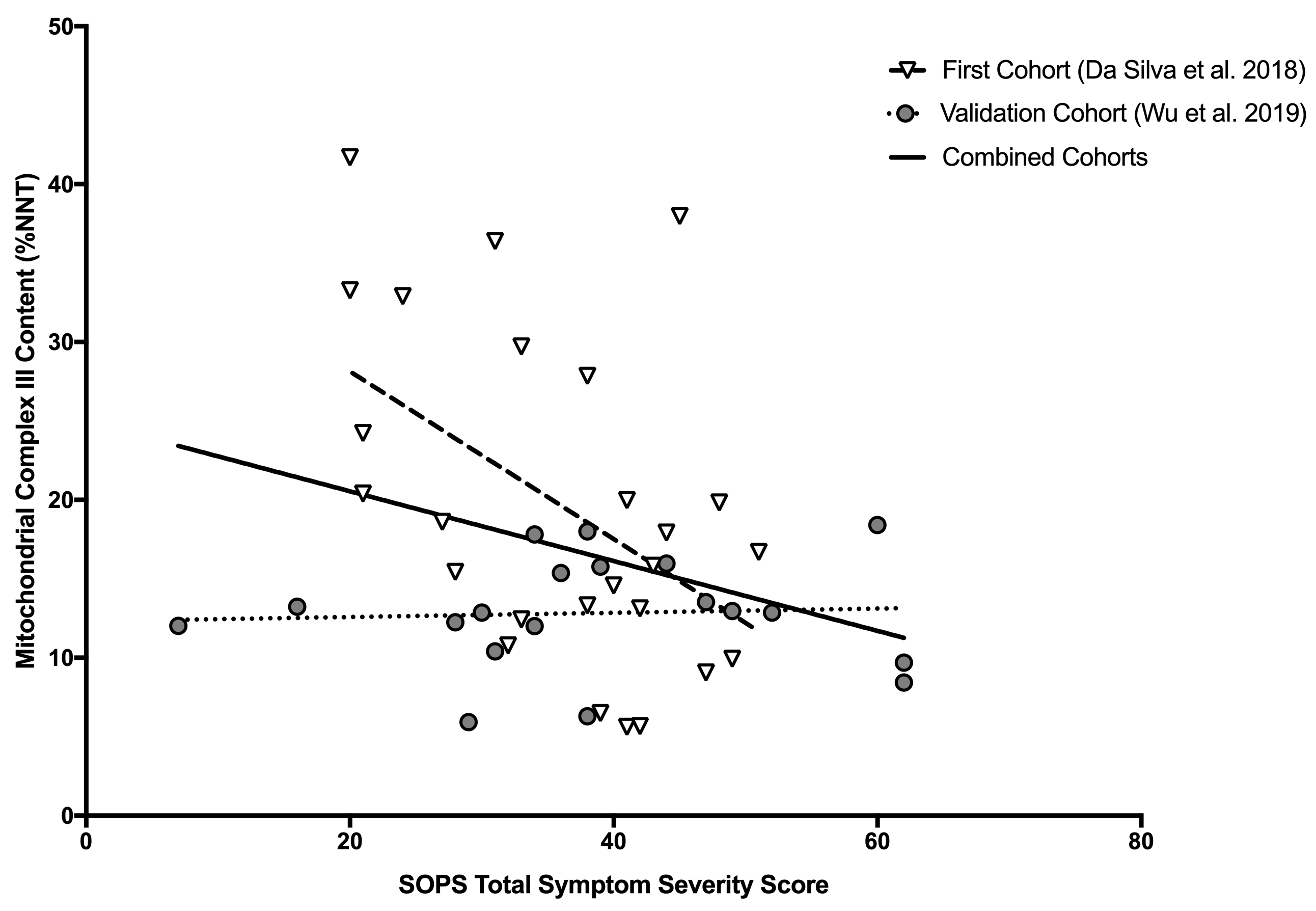


Figure S2: The comparison of bivariate correlation analyses between the first cohort, the second cohort, and the combination of the cohorts; examining the relationship between mitochondrial complex III content and SOPS total symptom severity score in the clinical high risk for psychosis group. The effect sizes are |r| = 0.49, |r| = 0.54, and |r| = 0.30 respectively.


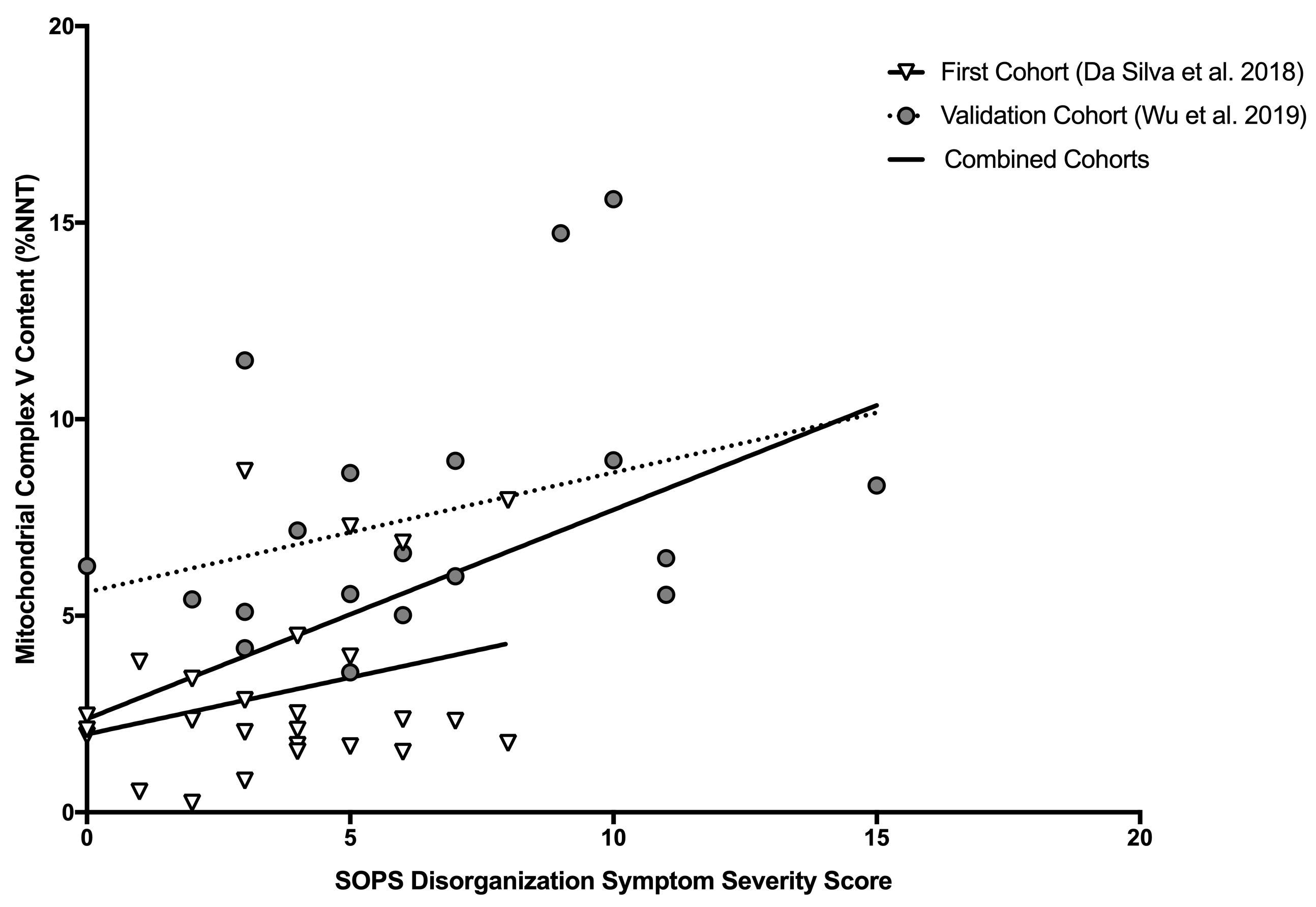


Figure S3: The comparison of bivariate correlation analyses between the first cohort, the second cohort, and the combination of the cohorts; examining the relationship between mitochondrial complex V content and SOPS disorganization symptom severity score in the clinical high risk for psychosis group. The effect sizes are |r| = 0.30, |r| = 0.35, and |r| = 0.50 respectively.


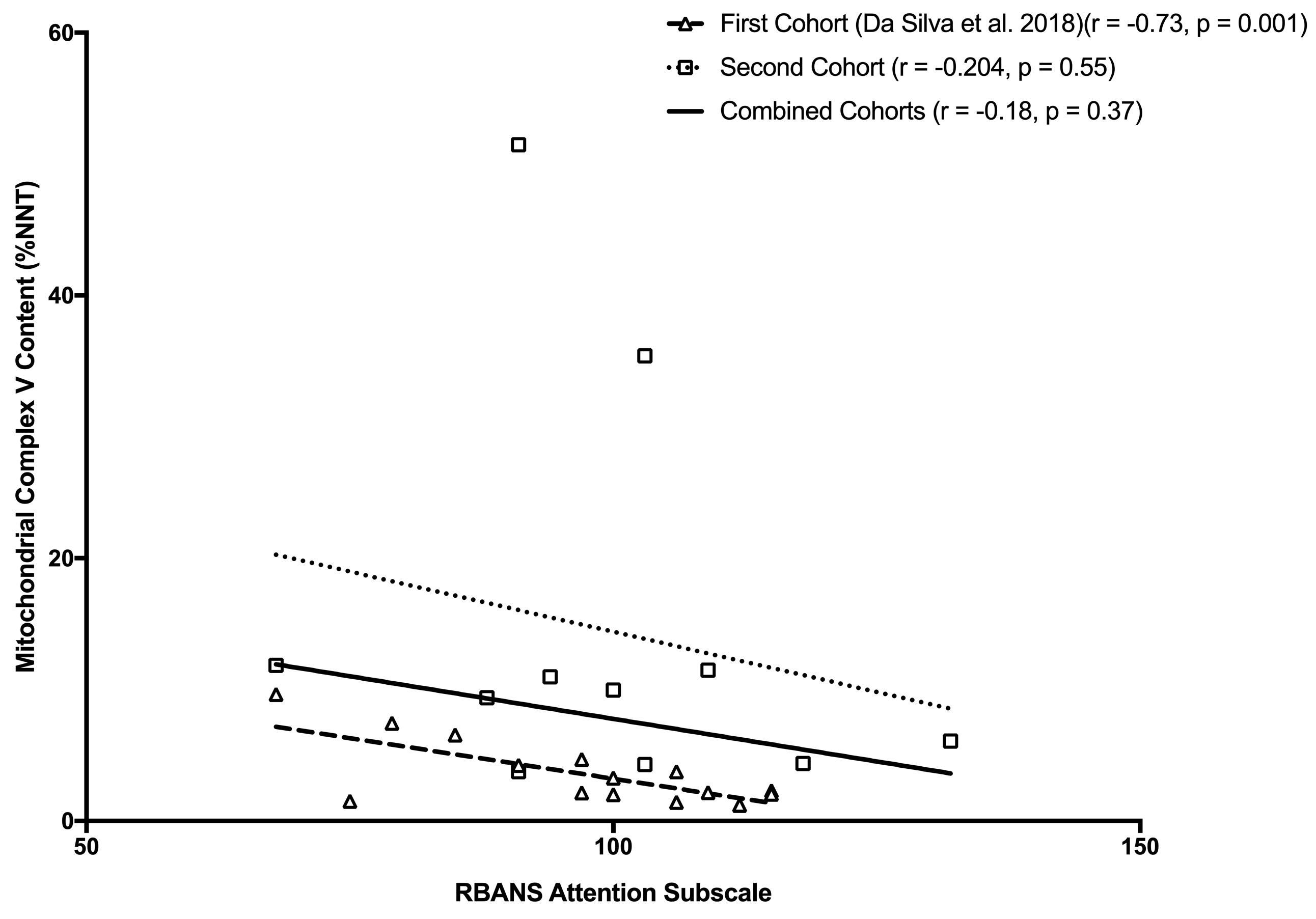


Figure S4: The comparison of bivariate correlation analyses between the first cohort, the second cohort, and the combination of the cohorts; examining the relationship between mitochondrial complex V content and RBANS Attention Subscale in the non-psychiatric control group. The effect sizes are |r| = 0.73, |r| = 0.55, and |r| = 0.18 respectively.


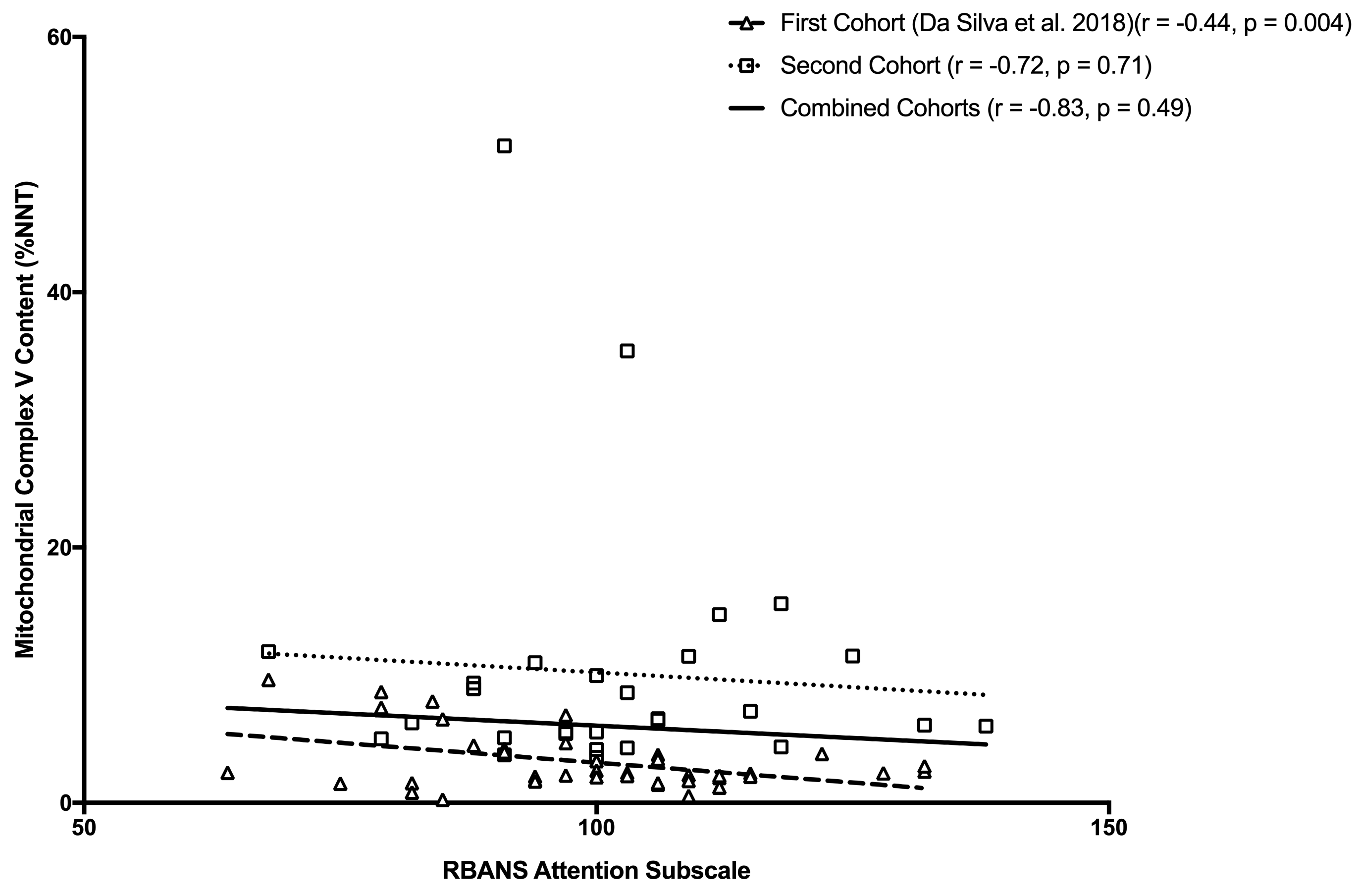


Figure S5: The comparison of bivariate correlation analyses between the first cohort, the second cohort, and the combination of the cohorts; examining the relationship between mitochondrial complex V content and RBANS Attention Subscale in both the clinical high risk for psychosis and non-psychiatric control groups. The effect sizes are |r| = 0.44, |r| = 0.44, |r| = 0.71, and |r| = 0.83 respectively.
